# Organic solvent tolerant metallo protease of novel isolate *Serratia marcescens* PPB-26: production and characterization

**DOI:** 10.1007/s13205-016-0500-0

**Published:** 2016-08-26

**Authors:** Shikha Thakur, Nirmal Kant Sharma, Neerja Thakur, Savitri, Tek Chand Bhalla

**Affiliations:** Department of Biotechnology, Himachal Pradesh University, Summer Hill, Shimla, Himachal Pradesh 171005 India

**Keywords:** *Serratia marcescens*, Organic solvent tolerant protease, Metalloprotease, 16S rDNA sequencing, Response surface methodology

## Abstract

Proteases are a class of enzymes that catalyze hydrolysis of peptide bonds of proteins. In this study, 221 proteolytic bacterial isolates were obtained by enrichment culture method from soils of various regions of Himachal Pradesh, India. From these a hyper producer of protease was screened and identified by morphological and physiological testing and by 16S rDNA sequence as *Serratia marcescens* PPB-26. Statistical optimization of physiochemical parameters enhanced the protease production by 75 %. Protease of *S. marcescens* PPB-26 was classified as a metalloprotease. It showed optimal activity at 30 °C, pH 7.5 (0.15 M Tris–HCl buffer) and with 0.8 % substrate concentration. It had *K*_m_ = 0.3 %, *V*_max_ = 34.5 μmol min^−1^ mg^−1^ protein and a half life of 2 days at 30 °C. The enzyme was stable in most metal ions but showed increased activity with Fe^2+^ and Cu^2+^ while strong inhibition with Co^2+^ and Zn^2+^. Further investigation showed that the enzyme could not only retain its activity in various organic solvents but also showed increased activity with methanol and ethanol. The reported metalloprotease is thus a potential candidate for carrying out industrial peptide synthesis.

## Introduction

Proteases, also known as peptidases belong to a larger group of enzymes called hydrolases which catalyze hydrolysis of bonds with the participation of a water molecule. They can be exoproteases or endoproteases depending on their site of action. On the basis of mechanism of catalysis, endoproteases are divided into aspartic proteases, cysteine proteases, metalloproteases, serine proteases and threonine proteases (Barrett et al. 2001). Metalloproteases are most diverse of all proteases (Barrett and Rawlings 1995). They require a divalent metal ion for their activity and are, therefore, inhibited by chelating agents such as EDTA (Miyoshi and Shinoda 2000). The metal ion acts by activating a water molecule which serves as nucleophile in the catalysis. Most metalloproteases contain Zn^2+^ while a few contain Mg^2+^, Ni^2+^ or Cu^2+^. Various microbial strains such as *Bacillus subtilis, Bacillus megaterium, Bacillus cereus, B. thuringiensis* *, Listeria monocytogenes, Serratia marcescens* AP3801 and *Serratia* sp. KCK have been reported to produce metalloproteases (Morita et al. 1997; Miyoshi and Shinoda 2000; Kim et al. 2007).

Proteases execute a large variety of complex physiological functions and their importance in conducting the essential metabolic and regulatory functions is evident from their occurrence in all living organisms. However, microbes are their most preferred source due to their rapid growth, limited space requirement and ease in genetic manipulation (Kocher and Mishra 2009). Among microbes, bacteria are the most common source of commercial proteases (Gupta et al. 2002). Bacterial proteases are mostly extracellular, easily produced in large amounts, thermostable and active at wide pH range. These properties make them most suitable for wide industrial applications. Due to the growing market of proteases worldwide efforts for isolation of novel bacterial species from underexploited regions and niche habitats that produce proteolytic enzymes with novel properties suitable for industrial applications, are underway.

Industrially, proteases are one of the most important categories of enzymes. They find application in variety of industries such as detergents, leather, food, textile and pharmaceutical industries (Ajithkumar et al. 2003; Bhaskar et al. 2007; Jellouli et al. 2009; Annapurna et al. 2012). Besides this, they are also used in waste treatment, peptide synthesis, diagnostic reagents and silver recovery from X-ray/photographic films (Rao et al. 1998; Bhalla et al. 1999; Upadhyay et al. 2010). Enzymatic peptide synthesis has attracted a great deal of attention in recent years. Hydrolysis of peptide bond by proteases in an aqueous environment is a reversible process and can be made to proceed towards peptide bond synthesis under water restricted environment. It has several advantages over chemical methods due to stereospecificity of the proteases, side-chain protection, non-toxic nature of solvents and recyclability of reagents (Morihara 1987). Most proteases, however, are inactive or show low activity in non-aqueous media (Vulfson et al. 2001). Several strategies have been employed to increase enzyme stability in non-aqueous environment such as protein engineering (Wolff et al. 1996) co-lyophilization with inorganic salts (Ru et al. 2000), chemical modification of amino acids on enzyme surfaces (Davis 2003), using supercritical fluids (Davis 2003) and using ionic liquids (Noritomi et al. 2009) However, to screen for naturally evolved solvent tolerant enzymes is more economical and cost effective. Thus, finding solvent stable protease has made an extensive area of research.

The present communication reports isolation of a novel protease source, i.e., *S. marcescens* PPB-26 from previously unexplored regions of Himachal Pradesh (H.P), statistical optimization of protease production and its characterization for potential in industrial applications.

## Materials and methods

### Chemicals

Different media components were obtained from Hi-Media (Mumbai, India). All other chemicals used were of analytical grade and procured from standard companies.

### Sample collection

Soil samples for isolation of proteolytic bacteria were collected aseptically from various regions of Himachal Pradesh (Shimla, Kullu, Manikaran, Manali, Kinnaur and Bilaspur) from sites that were rich in decaying garden waste, farm waste and industrial effluents. The samples were collected in sterile crew capped tubes and stored at 4 °C for further processing. The samples were processed within 24 h of collection.

### Isolation of bacteria for protease activity

Enrichment culture technique was used for isolation of proteolytic bacteria. One gram of soil samples were added into 50 ml minimal salt medium (MSM) supplemented with 1 % casein. Composition of the MSM broth (g/l) was: glucose, 1; peptone, 10; yeast extract, 0.2; CaCl_2_, 0.1; K_2_HPO_4_, 0.5; MgSO_4_, 0.1 and casein, 10, pH 7. The culture was grown for 24 h at 155 rpm in 30 °C temperature. Subsequent enrichments were done and after the third enrichment 1 ml culture broth was serially diluted to 10^−4^–10^−6^ times with physiological saline. The diluted inoculum (0.1 ml) was then plated on nutrient agar plates and incubated for 48 h at 30 °C. Bacterial colonies obtained were purified by sub-culturing and then preserved in nutrient agar slants kept at 4 °C.

### Screening of bacterial isolates for protease activity

Primary screening was done by streaking the isolates on 1 % skim milk agar plates and incubating overnight at 30 °C. Formation of clear zones around colonies indicated digestion of the milk protein and hence extracellular protease activity. Isolates showing strong protease activity were subjected to secondary screening by qualitative determination of their protease activity.

### Assay of protease activity

Protease activity in the isolates was assayed using the method developed by Manachini et al. (1988). Test reaction contained 4 ml of substrate solution, i.e., 0.5 % w/v casein in 50 mM potassium phosphate buffer (pH 7) and 1 ml of enzyme, while in control experiment no enzyme was added. Both reactions were incubated at 30 °C and then stopped after 20 min by adding 5 ml of 5 % w/v trichloro acetic acid and vortexed. They were then centrifuged (10,000×*g* for 5 min) and absorbance of supernatants measured by spectrophotometer at 275 nm.

### Characterization and strain identification

Physiological and biochemical characterization of the selected isolate PPB-26 was performed as per Bergey’s manual, using HiIMViCTM Biochemical Test Kit (Code no.: KB001) (HiMedia Laboratories Pvt. Ltd). Preliminary testing involved gram staining, spore formation, acid production tests (from glucose, adonitol, arabinose, lactose, sorbitol, mannitol, rhamnose and sucrose), indole test, methyl red test, Voges Proskauer test and citrate utilization test. Final identification was done by 16S rDNA gene sequence analysis. Genomic DNA of the selected isolate was extracted and 16S rDNA gene amplified by PCR using universal primers 27 F (5′-AGA GTT TGA TCC TGG CTC AG-3′) and 1541 R (5′-AAG GAG GTG ATC CAG CCG CA-3′). PCR products were purified and sequenced by Microbial Type Culture Collection and Gene Bank (MTCC), Institute of Microbial Technology, Chandigarh, India. The related sequences were obtained from GenBank database [National Centre for Biotechnology Information (NCBI), USA] using BLAST search programme and then aligned using the CLUSTAL W programme. A phylogenetic tree was then finally constructed using MegAlign software.

### Optimization of culture parameters for protease production

Following culture parameters were optimized using the OVAT (one variable at a time) approach.

### Selection of growth medium

Optimum growth media was selected by growing *S. marcescens* PPB-26 in twelve different media (M1–M12) which have previously been reported in literature. 50 ml of nutrient broth (pH 7) was used as seed media and the culture was incubated over night at 30 °C in an incubator shaker (155 rpm). 2 % of inoculum from the seed media was then added to 50 ml of M1–M12 production media which were further incubated in incubator shaker (155 rpm) at 30 °C. After 24 h the cells were harvested by centrifugation (10,000×*g* for 15 min) and the protease activity in their supernatant was assayed.

### Effect of carbon sources and its concentration

The effect of 12 different carbon sources on protease production was studied by supplementing 50 ml of the selected growth media with 2 % (w/v) of different carbon sources (starch, sucrose, maltose, mannitol, glycerol, lactose, galactose, fructose, dextrose, sodium acetate, sodium citrate and sodium succinate). The media were inoculated with 2 % inoculum from the seed media and incubated in incubator shaker (155 rpm) at 30 °C for 24 h. Protease activity in each was assayed as described in the preceding section. After selection of the suitable carbon source it was used in different concentrations (1–4 %) in the production medium to optimize its concentration.

### Effect of nitrogen sources and its concentration

To evaluate the effect of nitrogen sources on protease production, *S. marcescens* PPB-26 was grown in 50 ml each of production media supplemented with 1 % (w/v) of various organic and inorganic nitrogen sources (casein, yeast extract, beef extract, peptone, tryptone, soya meal, urea, ammonium sulphate, ammonium phosphate and ammonium nitrate). Incubation was done at 30 °C in an incubator shaker (155 rpm) for 24 h and then protease activity assayed. After selection of suitable nitrogen source its concentration was further optimized between 1 and 4 % in a manner similar to the preceding section.

### Effect of metal ions

Eight different metal ions were tested for their effect on protease production. 1 mM of metal ions (CaCl_2_·2H_2_O, CoCl_2_·6H_2_O, MgCl_2_·6H_2_O, MnCl_2_·6H_2_O, CuSO_4_·5H_2_O, MnSO_4_·H_2_O, FeSO_4_·7H_2_O and ZnSO_4_·7H_2_O) were added to 50 ml each of the production media. 2 % seed inoculum was added to these media and incubated at 30 °C for 24 h in an incubator shaker (155 rpm). Assays for protease activity were then carried out and the suitable metal ion selected.

### Incubation time

To find the optimum duration of incubation of *S. marcescens* PPB-26 for highest protease production, the organism was cultured in optimized growth conditions in an incubator shaker (155 rpm) at 30 °C for 56 h. Protease activity and cell growth in the media were recorded at regular intervals up to 56 h and the optimum incubation time was evaluated.

### Factorial design for statistical optimization of culture conditions

Design Expert software 9.0 was used to study the simultaneous effect of 11 independent variables on protease production by *S. marcescens* PPB-26. Using the software a Plackett–Burman design was generated having various combinations of high and low levels of the variables (pH, 5.0–9.0; temperature, 20–40 °C; dextrose, 0.5–5.0 %; tryptone, 0.1–2.0 %; casein, 0–2.0 %; yeast extract, 0.1–1.0 %; zinc sulphate, 0–0.01 %; potassium phosphate, 0–0.5 %; calcium chloride, 0–0.1 %; magnesium sulphate, 0–0.1 % and sodium chloride, 0–1.0 %). 12 different sets of experiments were performed in accordance with the design and protease activity in each case was assayed. The results were analyzed using the software and a Pareto chart was generated. From this chart, variables having positive effect on protease production were selected. Further a central composite design (CCD) was formed and 20 sets of experiments carried out to optimize concentration of the selected variables. The statistical model was validated for optimal production of protease by carrying out the experiment in a shake flask under the predicted set of conditions.

### Optimization of reaction conditions for the protease assay

#### Buffer system

To find out the optimum pH for protease activity, the reaction was carried out in various buffers of different pH at 30 °C for 20 min. Various buffer systems (citrate buffer, pH 4.0–6.0; Tris–HCl buffer, pH 7.0–9.0; potassium phosphate buffer, pH 6.0–8.0 and acetate buffer, pH 4.0–6.0) were used at a concentration of 0.1 M. After selection of the buffer, its molarity was optimized in the range of 25–200 mM.

#### Reaction temperature

Reaction temperature for the optimal activity of protease was found by carrying out reaction assays in 0.15 M Tris–HCl buffer (pH 7.5) at different temperatures (25–50 °C).

#### Substrate concentration

The effect of substrate (casein) concentration on the activity of protease of *S. marcescens* PPB-26 was studied by varying it in the range of 0.1–1.0 % in the reaction mixture.

#### Reaction time

To evaluate the optimum reaction time for activity of the crude protease, assay was carried out at different incubation times (2.5–60 min).

#### Effect of metal ions

The effect of various metal ions (CoCl_2_, FeCl_2_, MnCl_2_·4H_2_O, CuSO_4_·5H_2_O, FeSO_4_·7H_2_O, MnSO·H_2_O, ZnSO_4_·7H_2_O, MgSO_4_·7H_2_O and CaCl_2_·2H_2_O) on protease activity was tested by carrying out the reaction in the presence of 2 mM concentration of metals ions, at 30 °C for 10 min.

#### Effect of NaCl

The protease assay of *S. marcescens* PPB-26 was carried out in the presence of different concentrations of NaCl (0.1–0.9 M) and its effect on the enzyme activity was studied.

#### Effect of organic solvents

The crude protease enzyme was pre-incubated with 50 % (v/v) of various organic solvents. The effect of these organic solvents (acetone, acetonitrile, benzene, butanol, ethanol, isopropyl alcohol and methanol) on enzyme stability was studied by assaying the protease activity.

## Results

### Morphological and biochemical characteristics

Two hundred and twenty-one bacterial isolates were obtained from different soil samples. Isolate PPB-26, which emerged as a hyper producer of protease among all isolates was found to be a Gram negative, straight rod shaped, non-spore forming microbe. It shows negative results with indole test, methyl red test and Voges Proskauer test but was positive for citrate utilization. Further it was capable of glucose, adonitol, arabinose, sorbitol, mannitol and sucrose utilization but showed negative results for lactose and rhamnose.

## 16S rDNA gene sequencing

**Fig. 1:**
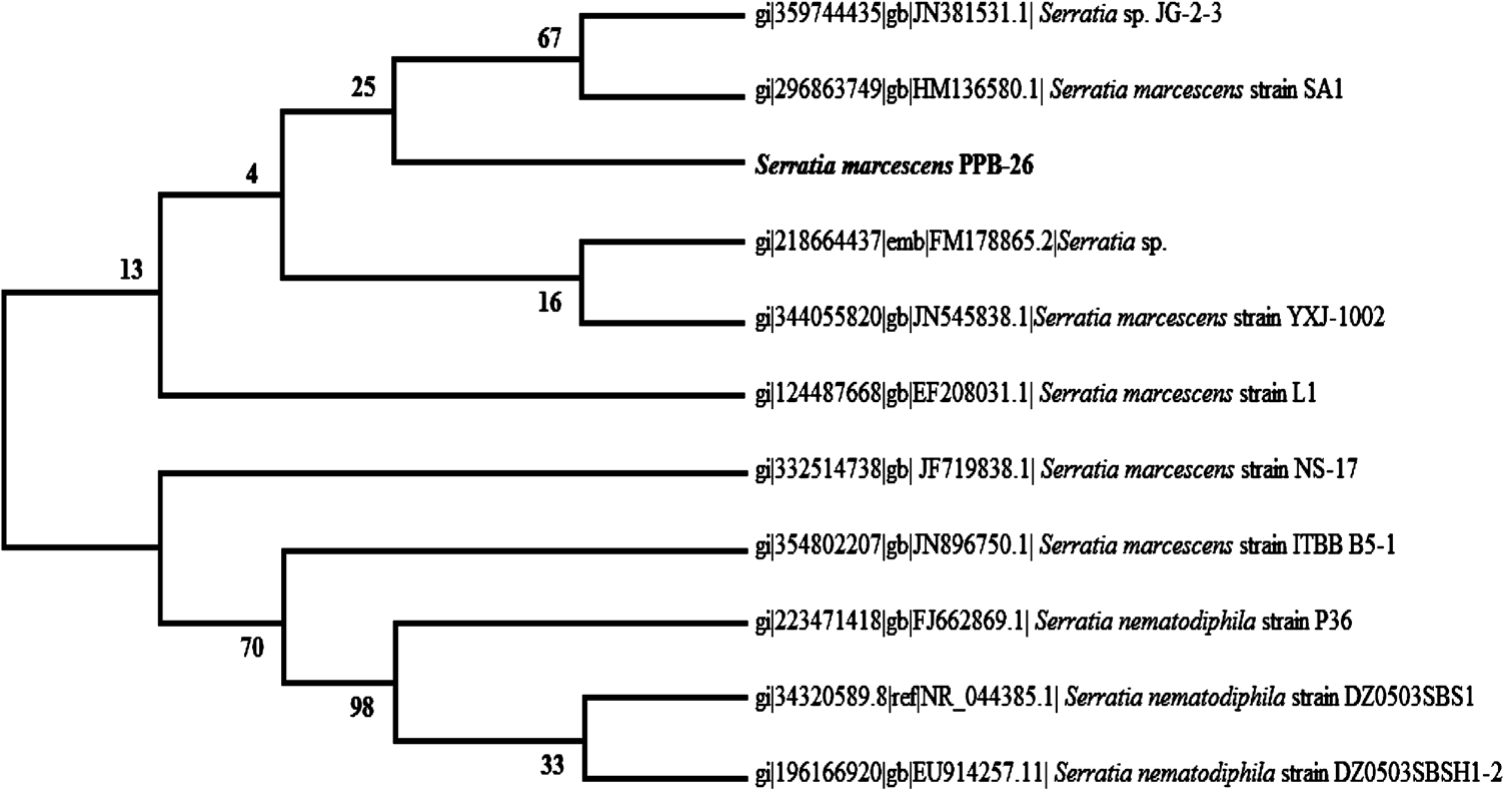
Phylogenetic dendrogram based on the 16S rDNA gene sequences

The sequence of isolate PPB-26, obtained by 16S rDNA gene sequencing was deposited in the NCBI GenBank database with accession number KJ735909. Phylogenetic analysis (Fig. 1) of this sequence revealed 99 % sequence similarity with *S. marcescens* strain SA1 (HM136580.1) indicating that isolate PPB-26 was closely related to it. Thus, on the basis of physiological and biochemical characteristics and on comparison of its 16S rDNA sequence the strain was identified as *S. marcescens* and named as *S. marcescens* PPB-26.

### Optimization of Culture Parameters for Protease Production by *S. marcescens* PPB-26

#### Media optimization

*Serratia marcescens* PPB-26 was cultured in 12 different media (Table 1) and medium M2 containing (g/l) 20.0 g glucose, 4.0 g yeast extract, 1.5 g potassium phosphate, 0.5 g magnesium sulphate, 0.5 g calcium chloride and 10 g casein proved to be the best media for protease production since in it, highest protease activity was observed (9 U/mg protein).

**Table 1:** Different media used for protease production by *S. marcescens* PPB-26

Medium code	Specific activity (U/mg of protein)	References
M1	8.1	GYP medium
**M2**	9	GYC medium
M3	5	Sinha and Satyanarayana (1991)
M4	0.3	Chu et al. (1992)
M5	5.6	Nutrient casein broth
M6	3	Tsuchiya et al. (1991)
M7	8.1	Tsujibo et al. (1990)
M8	8.7	Matta et al. (1997)
M9	8.6	Purva et al. (1998)
M10	8.7	Tsuchida et al. (1986)
M11	8.5	Kobayashi et al. (1995)
M12	8.1	Minimal medium

#### Carbon source

Among various carbon sources maximum protease activity (9.02 U/mg protein) was recorded in media supplemented with dextrose. The concentration of dextrose was further optimized and 2.5 % was found to be the most suitable.

#### Nitrogen source

Maximum protease activity (9.4 U/mg protein) was observed in media containing tryptone as the nitrogen source. Optimum concentration of tryptone was found to be 1.5 %. Protease production by *S. marcescens* PPB-26 was higher in organic nitrogen sources than in inorganic sources.

#### Metal ions

In media supplemented with 1 mM CaCl_2_ the highest protease activity (10 U/mg protein) was observed. However, with metal ions MnCl_2_ (4 U/mg protein) and MnSO_4_ (8.5 U/mg protein) an inhibition in enzyme activity was recorded.

#### Incubation time

The activity profile of *S. marcescens* PPB-26 protease studied over a growth period of 56 h shows a sharp increase in protease activity after 12 h, i.e., during exponential growth phase of *S. marcescens* PPB-26. Maximum protease activity (10.03 U/mg protein) was recorded at 24 h of incubation. There after the protease activity stayed stable till 54 h and later declined gradually.

### Statistical optimization

**Fig. 2:**
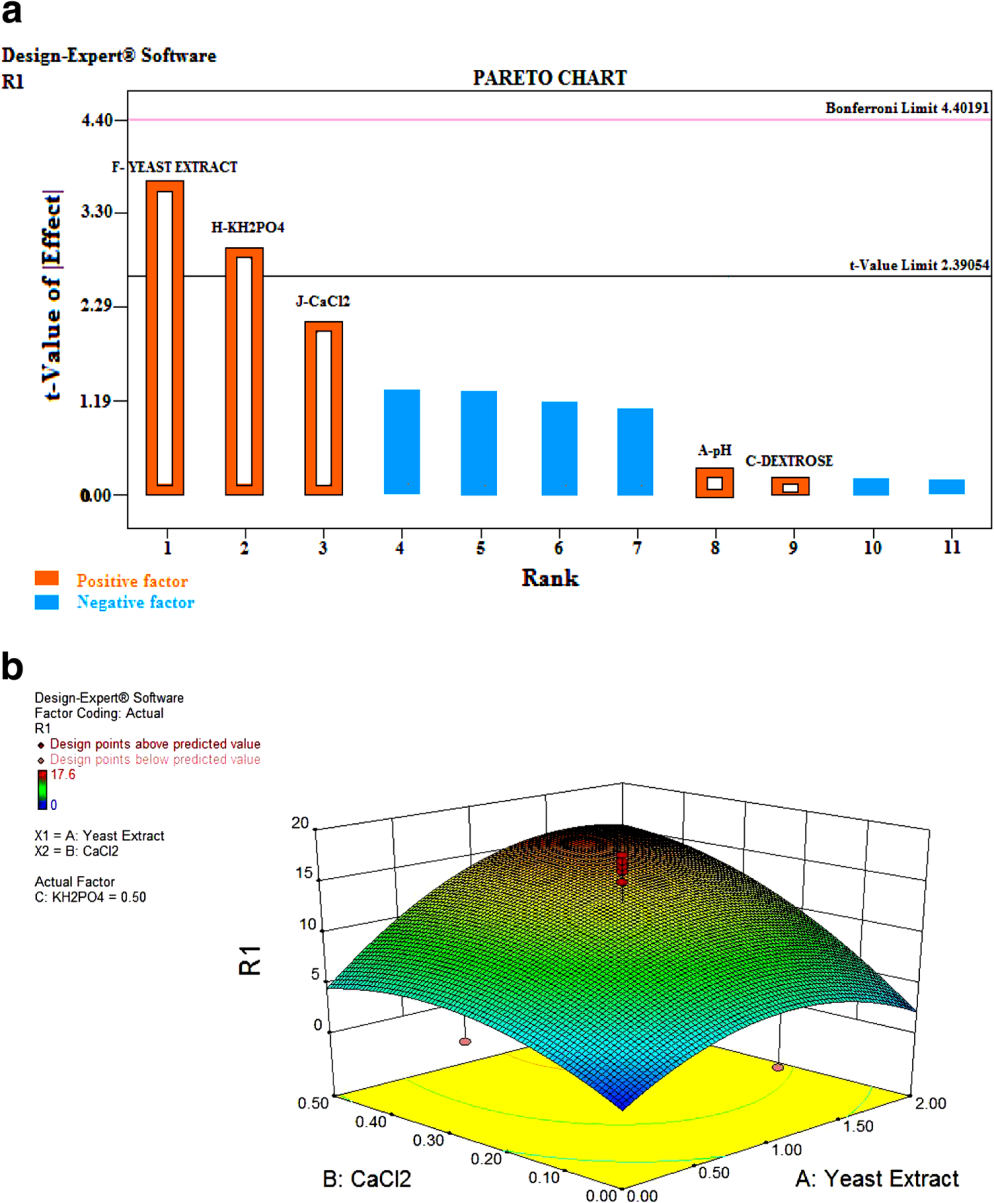

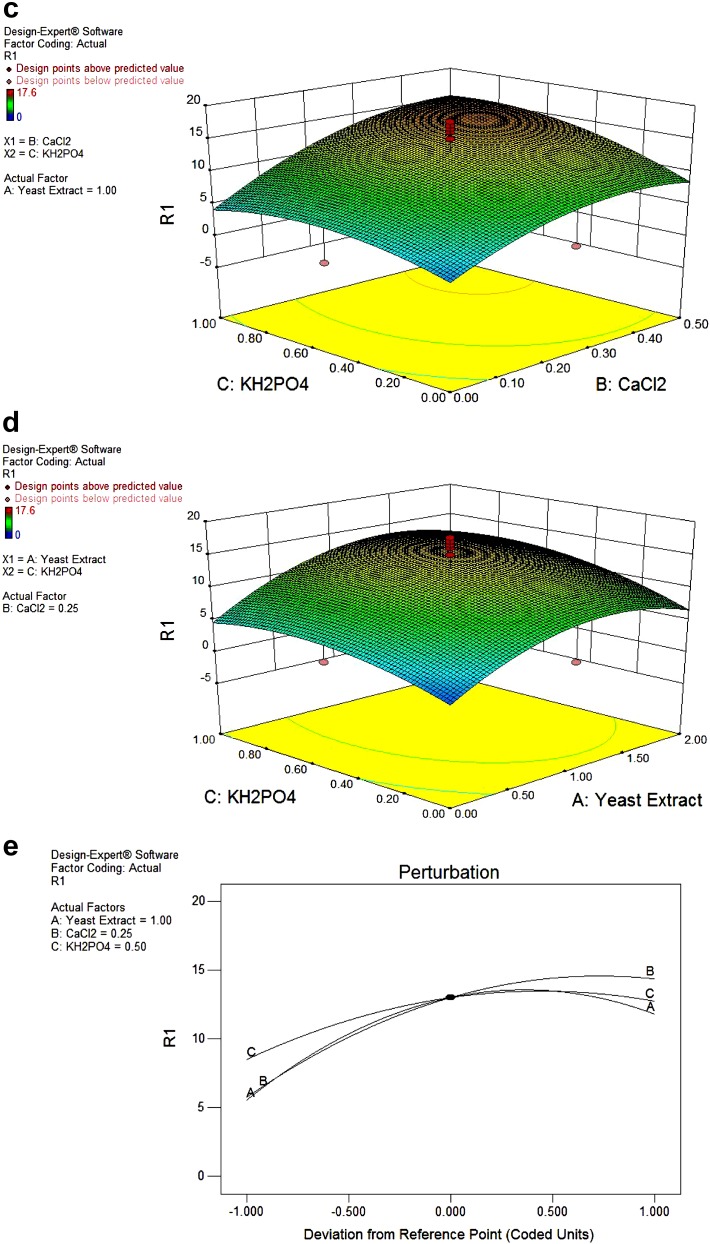
**a** Pareto chart showing positive and negative factors for protease production by *S. marcescens* PPB-26. **b** Three dimensional response surface plot for the effect of (*A*) CaCl_2_ and (*B*) Yeast extract on protease production by *S. marcescens* PPB-26. **c** Three dimensional graph showing effect of two variable interaction (*A*) KH_2_PO_4_ and (*C*) CaCl_2_ on protease production. **d** Three dimensional (3D) graph showing effect of two variable interaction effect (*B*) KH_2_PO_4_ and (*C*) Yeast extract on protease production. **e** Perturbation plot showing the optimum value for optimized variables

Results of experiments performed in accordance with the Plackett–Burman design are shown in Table 2. Highest protease activity obtained from this design was 9.8 U/mg protein. Using the responses from these set of experiments a Pareto Chart (Fig. 2a) was generated which shows the order of significance of variables on protease production by *S. marcescens* PPB-26. The chart showed that yeast extract, potassium phosphate and calcium chloride had the greatest positive effect on protease production. A central composite design was thus generated taking different combinations of these three variables. Twenty different sets of experiments were performed according to this design (Table 3) varying the concentrations of these three variables while keeping the concentrations of all other variables constant (2.5 % dextrose, 0.5 % magnesium sulphate, 1 % casein, 1.5 % tryptone, pH 7.0 and temperature 30 °C). Highest protease activity was obtained in the set containing 1 % yeast extract, 0.5 % potassium phosphate and 0.25 % calcium chloride. Optimized concentrations of the three selected variables were further validated by three dimensional (3D) response surface plots (Fig. 2b–d) generated using the statistical software. These plots show the effects of independent variables as well their combined effect on the response, i.e., protease activity. They also show the predicted value of the protease activity at the optimized concentrations of the selected variables. The model was validated by performing the experiment under the predicted set of conditions indicated by the perturbation plot (Fig. 2e). The response obtained (17.5 U/mg protein) was very close to the predicted response (17.6 U/mg protein), thus the validity of the model was proved. Factorial design caused a 1.75 fold (75 %) increase in the production of protease by *S. marcescens* PPB-26.

**Table 2:** Plackett-Burman experimental design for production of proteolytic activity

Run	pH	Temperature (°C)	Dextrose (%)	Tryptone (%)	Casein (%)	Yeast Ext. (%)	ZnSO_4_ (%)	KH_2_PO_4_ (%)	CaCl_2_ (%)	MgSO4 (%)	NaCl (%)	Response (U/mg protein)
1	9.0	20	5.0	2.0	0	1.0	0.01	0.5	0	0	0	9.0
2	9.0	40	0.5	2.0	2.0	1.0	0	0	0	0.1	0	0
3	5.0	20	5.0	0.1	2.0	1.0	0	0.5	0.1	0.1	0	9.8
4	9.0	40	5.0	0.1	0	0.1	0.01	0	0.1	0.1	0	0
5	9.0	40	0.5	0.1	0	1.0	0	0.5	0.1	0	1.0	0
6	5.0	40	5.0	0.1	2.0	1.0	0.01	0	0	0	1.0	1.0
7	5.0	40	5.0	2.0	0	0.1	0	0.5	0	0.1	1.0	0
8	9.0	20	0.5	0.1	2.0	0.1	0.01	0.5	0	0.1	1.0	2.6
9	5.0	20	0.5	0.1	0	0.1	0	0	0	0	0	0
10	9.0	20	5.0	2.0	2.0	0.1	0	0	0.1	0	1.0	1.163
11	5.0	40	0.5	2.0	2.0	0.1	0.01	0.5	0.1	0	0	0.0078
12	5.0	20	0.5	2.0	0	1.0	0.01	0	0.1	0.1	1.0	7.0

**Table 3:** Central composite experimental design

Run	Yeast Extract (%)	CaCl_2_ (%)	KH_2_PO_4_ (%)	Response (U/mg protein)
1	1.0	0.25	0.5	4.9
2	0	0.25	0.5	3.0
3	0.02	0	0.5	0
4	2.67	0.25	1.0	5.3
5	1.0	0.25	0.5	16
6	1.0	0.25	0.5	15
7	0.02	0.5	1.0	4.0
8	2.0	0.5	1.0	17
9	1.0	0.25	0	3.0
10	1.0	0.25	1.34	10
11	1.0	0	0.5	0.5
12	1.0	0.25	0.5	16.5
13	0.2	0	0	0
14	1.0	0.25	0.5	17
15	1.0	0.67	0.5	12
16	2.0	0	1.0	1.0
17	2.0	0	0	0.8
18	0.02	0.5	0	1
19	1.0	0.25	0.5	17.6
20	2.0	0.5	0	9

### Optimization of reaction conditions

#### Buffer system and pH

1.5 M tris–HCl buffer at pH 7.5 was found to be the most appropriate buffer (17.7 U/mg protein) for protease activity of *S. marcescens* PPB-26 protease.

#### Reaction temperature

Maximum enzyme activity (17.8 U/mg protein) was recorded at 30 °C. The proteolytic activity stayed stable till 40 °C, but beyond it there was a steady decline in activity.

#### Substrate concentration

The effect of substrate (casein) concentration (0.1–1.0 %) on protease activity of *S. marcescens* PPB-26 was studied and maximum activity (20 U/mg protein) was observed at 0.8 % casein. At higher concentrations decline in activity was recorded.

#### Determination of *K*_m_ and *V*_max_

**Fig. 3:**
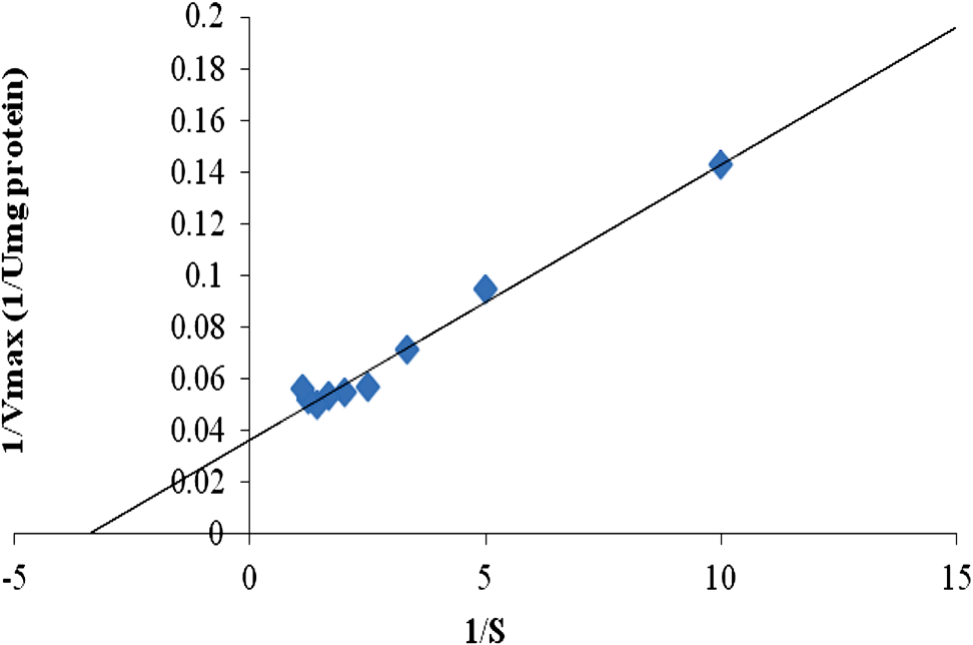
Lineweaver-Burk plot for the protease of *S. marcescens* PPB-26

The protease of *S. marcescens* PPB-26 followed normal Lineweaver–Burk plot up to 0.3 % casein. The analysis of Lineweaver–Burk plot showed that the protease had *K*_m_ = 0.3 % and *V*_max_ = 34.5 μmol min^−1^ mg^−1^ protein (Fig. 3).

#### Reaction time

For optimizing reaction time, protease assay was carried out for 60 min and tyrosine produced in the reaction mixture was recorded at regular interval. Ten minutes was found to be the shortest duration during in which highest tyrosine concentration (35 µg/ml) is produced. Beyond this the rate of increase in tyrosine concentration remained same.

#### Effect of metal ions

Co^2+^ (16.6 U/mg protein) and Mn^2+^ (18 U/mg protein) inhibited the protease activity, whereas Fe^2+^ (22.5 U/mg protein) and Cu^2+^ (21.8 U/mg protein) had an incremental effect. Other metal ions (Ca^2+^, Cu^2+^, Zn^2+^ and Mg^2+^) had no major effect on the protease activity.

#### Effect of NaCl

The protease activity of *S. marcescens* PPB-26 stayed stable (17 U/mg protein) with up to 0.9 M concentration of NaCl.

#### Stability in organic solvents

**Fig. 4:**
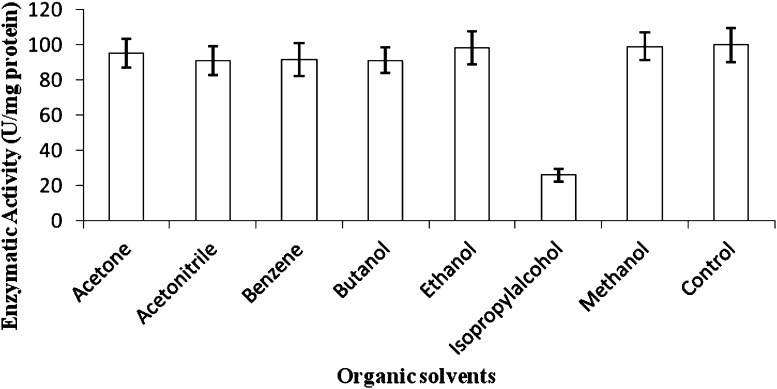
Effect of organic solvents on protease activity of *S. marcescens* PPB-26

The protease enzyme was found to be stable in most organic solvents (Fig. 4) except in isopropanol in which the activity was severely reduced. Maximum stability in the protease activity was recorded with methanol (99 %) and ethanol (96 %).

## Discussion

Application of enzymes instead of chemicals in the industry is preferred as it provides for a cheaper and more environment friendly alternative. This study was focused on isolation and screening of bacteria from soils of Himachal Pradesh for high protease activity. Soil has served as source of numerous protease producing bacteria such as *B. megaterium* (Asker et al. 2013). After exhaustive screening a hyper producer of protease was selected which phenotypically resembled the earlier reported *S. marcescens* strain SA1 (HM136580.1). The isolate *S. marcescens* PPB-26 showed highest protease activity in GYC medium while lowest in M4 medium (soybean meal medium). Soybean has anti-trypsin inhibitors which reduce the protein digesting capability (Haard et al. 1996). Among different carbon sources, glucose was the most preferred while sucrose, fructose and galactose were least preferred for protease production by *S. marcescens* PPB-26. Glucose has previously been reported as a suitable carbon source for protease production in *S. marcescens* (Mohankumar 2011). Joshi et al. (2007) reported fructose as preferred carbon source for protease production by *B. cereus* MTCC 6840. Among nitrogen sources, organic sources were better for protease production than inorganic sources which resulted in decreased protease activity. A similar trend has been reported in serration peptidase enzyme of *S. marcescens* (Mohankumar 2011). Of different metal ions studied most showed no effect on enzyme activity except manganese chloride which had an inhibitory effect and calcium chloride which increased protease activity. Metal ions stabilize and enhance the production and activity of protease enzyme (Janssen et al. 1994). Calcium has been reported to enhance protease production in *Pseudomonas aeruginosa* (Marquart et al. 2005). Not many reports of statistical optimization of protease production have been reported. In this study *S. marcescens* PPB-26 protease production was optimized using the Response Surface Methodology. Factorial designing increased the production by 1.75 fold (75 %). In *P. aeruginosa* MCM B3271.3 fold increase was reported (Zambare et al. 2013).

*Serratia marcescens* PPB-26 protease showed activity in pH range of 6–9 with maximum activity at pH 7.5 in 1.5 M Tris–HCl buffer. Ionic strength of buffer did not have much effect on the enzyme activity. Optimum temperature for enzyme activity was found to be 30 °C, at higher temperatures beyond 40 °C the activity decreased. The enzyme faced product inhibition at concentrations above 0.8 % casein due to product accumulation in the reaction mixture. Among metal ions Co^2+^, Zn^2+^ and Mn^2+^ had negative effect on the protease activity, whereas Fe^2+^ had a positive effect on it. Similar results with metal ions have been reported previously also (Yang et al. 2000). The protease of *S. marcescens* PPB-26 showed tolerance to high (0.9 M) NaCl concentrations. Decrease in activity at higher NaCl concentrations might be due to the precipitation of substrate at high salinities. The protease of *Bacillus* sp. NG-27 has also been reported to exhibit high salt tolerance (Sumandeep et al. 1999).

Organic solvents are used as reaction media for enzymes in various industrial processes due to advantages like high regio- and stereo-selectivity and minimal side-chain protection requirements (Kumar and Bhalla 2005). However, most enzymes show low activity in organic solvents due to limited diffusion, partial denaturation and reduced flexibility (Kwon et al. 1999). The protease of *S. marcescens* PPB-26 showed stability in all organic solvents (50 % v/v) over 24 h, except in isopropanol. This property makes it useful for industrially important applications like peptide synthesis. The enzyme showed maximum stability (95–100 %) with toxic organic solvents like methanol and ethanol, unlike the previously reported proteases of *B. tequilensis* P15 Bose et al. 2014) and *Virgibacillus* sp. EMB13 (Sinha and Khare 2012) in which the relative activity was 2 % (in methanol) and 45 % (in ethanol), respectively.

## Conclusion

This study reports an organic solvent tolerant protease from *S. marcescens* PPB-26 which shows activity in a broad range of temperature and pH. Stimulation of proteolytic activity in presence of CaCl_2_ is an interesting characteristic as very few reports of such effects exist in literature. The enzyme’s stability in the presence of a wide range of organic solvents, metal ions as well as salinity indicates its huge potential in various industrial applications such as peptide synthesis and as a detergent additive.
